# TAM-targeted reeducation for enhanced cancer immunotherapy: Mechanism and recent progress

**DOI:** 10.3389/fonc.2022.1034842

**Published:** 2022-11-07

**Authors:** Xinyuan Shen, Shengcheng Zhou, Yidong Yang, Tu Hong, Ze Xiang, Jing Zhao, Chaojie Zhu, Linghui Zeng, Lingxiao Zhang

**Affiliations:** ^1^ Key Laboratory of Novel Targets and Drug Study for Neural Repair of Zhejiang Province, School of Medicine, Zhejiang University City College, Hangzhou, China; ^2^ College of Pharmaceutical Sciences, Zhejiang University, Hangzhou, China; ^3^ Key Laboratory of Diagnosis and Treatment of Digestive System Tumors of Zhejiang Province, Ningbo Hwa Mei Hospital, University of Chinese Academy of Sciences, Ningbo, China; ^4^ Ningbo Institute of Life and Health Industry, University of Chinese Academy of Sciences, Ningbo, China

**Keywords:** tumor microenvironment, tumor-associated macrophages, TAM reeducation, nanomedicine, cancer immunotherapy

## Abstract

Tumor-associated macrophage (TAM) as an important component of tumor microenvironment (TME) are closely related with the occurrence, development, and metastasis of malignant tumors. TAMs are generally identified as two distinct functional populations in TME, *i.e.*, inflammatory/anti-tumorigenic (M1) and regenerative/pro-tumorigenic (M2) phenotype. Evidence suggests that occupation of the TME by M2-TAMs is closely related to the inactivation of anti-tumor immune cells such as T cells in TME. Recently, efforts have been made to reeducate TAMs from M2- to M1- phenotype to enhance cancer immunotherapy, and great progress has been made in realizing efficient modulation of TAMs using nanomedicines. To help readers better understand this emerging field, the potential TAM reeducation targets for potentiating cancer immunotherapy and the underlying mechanisms are summarized in this review. Moreover, the most recent advances in utilizing nanomedicine for the TAM immunomodulation for augmented cancer immunotherapy are introduced. Finally, we conclude with our perspectives on the future development in this field.

## Introduction

Tumor microenvironment (TME) with high anti-inflammatory immune cell frequencies and an immunosuppressive network is the culprit responsible for the inefficiency of clinical cancer immunotherapy (1, 2). TME is a highly complex system composed of abnormal vasculatures, dense extracellular matrix (ECM), fibroblasts, tumor-resident immune cells (*e.g.*, tumor-associated myeloid cells, mast cells, natural killer (NK) cells, tumor-associated macrophages (TAMs), T and B lymphocytes), and various secreted factors (3, 4). In most cases, the anti-inflammatory TME forms immunosuppressive networks that results in immune resistance and ultimately facilitate tumor growth or metastasis (5). High levels of extracellular matrix, hypoxia, immunosuppressive cytokines, toxic metabolites, and high expression of immune checkpoint molecules are the main contributors of tumor immune suppression (6, 7). Meanwhile, different types of TME, which are composed of distinct species and content of immune cells are also closely related to clinical immunotherapy efficacy (8).

TAMs are important components of infiltrating immune cells in TME, most of which are differentiated from monocytes recruited from the periphery to the tumor site (9). Chemokines secreted by tumor cells or TAMs such as C-C motif chemokine ligand 1(CCL1), CCL2, CCL5, transforming growth factor-*β* (TGF-β), platelet-derived growth factor, vascular endothelial growth factor (VEGF), and colony-stimulating factor-1 (CSF-1) can recruit monocytes into TME and promote their differentiation into TAMs (10, 11). Meanwhile, resident macrophages surrounded by solid tumors such as lung or brain tumors may also proliferate *in situ* and infiltrate into TME (12). Besides, the downregulation of the transcription factor signal transducers and activators of transcription (STAT) 3 (STAT3) also promotes monocyte-associated bone marrow-derived suppressor cells to differentiate into mature TAMs (13).

Notably, the presence of TAMs in TME has been demonstrated to greatly restrict the therapeutic effects of chemotherapy and radiotherapy (14). In fact, TAMs exert double-sword effects in both promoting and inhibiting tumor growth according to their phenotype, which can be divided into M1 (inflammatory/anti-tumorigenic) or M2 (regenerative/pro-tumorigenic) type according to their surface molecules and functions. Usually, TAMs differentiate into M1 phenotype in the presence of interferon-γ (IFN-γ), tumor necrosis factor *α* (TNF-α), lipopolysaccharide (LPS), or granulocyte-macrophage colony-stimulating factor (GM-CSF) stimulation and are characterized by the subsequent activation of Toll-like receptor (TLR) signaling pathways (15, 16). In general, M1-TAMs exert cytotoxic effects on tumor cells by secreting nitric oxide or producing TNF-α, IFN-γ, or IL-12, and also triggers the activity of NK cells and prime cytotoxic T cells (17). In addition, M1-TAMs can effectively promote the level of tumor-infiltrating T cells by triggering an inflammatory response, which further confirms the role of M1-TAM in the regulation of pro-inflammatory TME (18). In contrast, M2-TAMs usually lead to an immunosuppressive TME by abolishing the activity of T cells through both direct and indirect pathways. The expression of arginase 1receptor (ARG1) in M2-like TAMs catabolizes arginine that are necessary for T cell activation and proliferation, thereby inhibiting T cell proliferation (19). At the same time, M2-TAMs also directly inhibit the cytotoxicity of CD8^+^ T cells or NK cells by releasing arginase 1, producing IL-10 and TGF-*β* or promote T regulator (Treg) proliferation (20). Besides, M2-TAMs-mediated CCL2, CCL3, CCL4, CCL5 or CCL20 release also promotes the recruitment of Treg cells into TME, which further suppresses the anti-tumor functions of T cells and NK cells (21).

**Figure 1:**
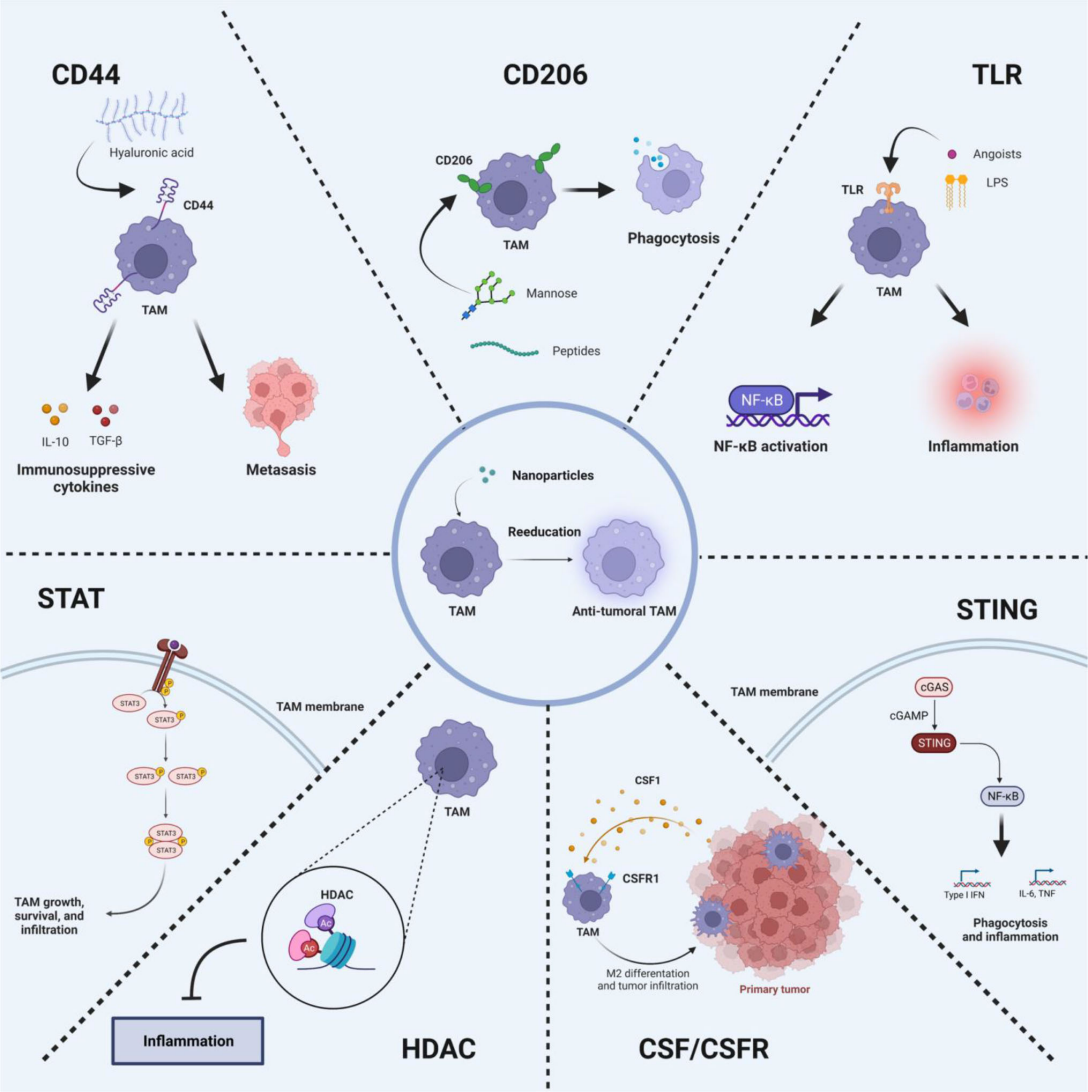
Signaling pathways for the precise reeducation of TAMs.

In light of these findings, strategies to reeducate TAMs from M2- to M1-phenotype has been a promising approach for potentiating cancer immunotherapy. TAMs polarization is a sophisticatedly regulated biological process, determined by several signaling cascades triggered by receptor engagements or intracellular regulatory proteins. TAMs stimulated by IFN-γ usually differentiate into typical M1-phenotype *via* the activating Toll-like receptor (TLR) pathway, while TAMs are polarized from M1- to M2-pheynotype at the presence of CSF-1, IL-4, IL-13 or IL-10 through Janus tyrosine Kinase (JAK)-STAT signaling pathway (22). Moreover, several signaling pathways also participate into the polarization of TAMs, including CD44, TLR, cGAS-STING, CSF1-CSF1R, CD206, STAT, and histone deacetylase (HDAC) pathways. Herein, the mechanism of these signaling pathways in reeducation of TAMs is systematically summarized, and the corresponding TAMs-targeted nanomedicines for enhancing cancer immunotherapy are also provided (**Figure 1**).

## Strategies to reeducate TAMs

The regulation of TAM is affected by various signaling pathways in TME, but the underlying mechanism has not been fully revealed. Notably, the polarization of TAMs is a highly plastic process, which is not strictly a bipolar system (23). Upon exposure to different stimuli in TME, TAMs may present hybrid phenotypes and exhibit complex characteristics. Therefore, we first discuss the effects of intracellular signaling pathways regulation on the biological behaviors of TAMs and anti-tumor therapeutic efficacy (24, 25). The enhanced permeability and retention effect-mediated tumor accumulation (26) and surface-ligand directed M2-like TAMs-targeting drug delivery (27) enable nanomedicines to effectively reeducate TAMs. Therefore, nanomedicines that can positively regulate TAMs to anti-tumor phenotype and amplify cancer immunotherapy are introduced according to the signaling pathways involved to TAMs reeducation.

### CD44 pathway

CD44 is a transmembrane adhesion glycoprotein mainly expressed on endothelial cells, mesenchymal cells, monocytes, neutrophils, and lymphocytes (28–30). Aberrant upregulation of CD44 in tumor cells is closely related to tumor immune escape and invasion, and involves reduced oxygen levels and regulation of HIF-1 signaling (31). CD44 is a biomarker of M2-TAMs, the level of which in glioma is closely associated with infiltration of M2-TAMs (32), and consistent with the CD116, CD14, CD68, and CD163 (33). Evidence suggests that CD44^+^ TAMs can activate angiogenesis by secreting VEGFR and promote tumor cell invasion by secreting matrix metallopeptidases (34, 35).

Hyaluronic acid (HA) secreted by cancer-associated fibroblasts and cancer cells is an abundant component of the ECM in TME, which is the main ligand of CD44 (36). Under physiological conditions, low molecular weight HA (LMW-HA) usually exhibits pro-inflammatory function, while the high molecular weight HA (HMW-HA) displays anti-inflammatory and anti-angiogenic effects (37). The interaction of LMW-HA with CD44 stimulates TLR signaling and exacerbates inflammatory responses (38). HA binds the CD44 ligand induces a conformational change in CD44, which in turn activates various signaling pathways (39). Meanwhile, the HA fragment upregulates the expression of CD44 and TLR4, activates NF-*κ*B translocation, and increases the secretion of TNF-*α*, IL-6, and IL-1*β* (40). Notably, the interaction of CD44 with HA is also regulated by a variety of cytokines such as IL-2, tumor necrosis factor (TNF), and chemokines, which activates CD44 to enhance the HA-CD47 interaction (41). A recent study shows that disruption of tumor HA synthesis or blocking HA binding to CD44 on TAM effectively increased the proportion of M1-TAMs by upregulating SIRPα (42). Besides, HA also binds to other receptors such as TLR4, which inhibit the lipopolysaccharide-induced activation of inflammatory macrophages or monocytes (43). Furthermore, the ERK1/2 signaling cascade is closely associated with HA and CD44 and is involved in the regulation of bone marrow differentiation (44). ERK1/2 activated by HA-CD44 interaction also induces phenotypic transformation of TAMs. Evidence shows that the inhibition of ERK1/2 signaling successfully blockades the STAT3 activation and thus restricting TAMs differentiate into M2-phenotype (45).

**Figure 2:**
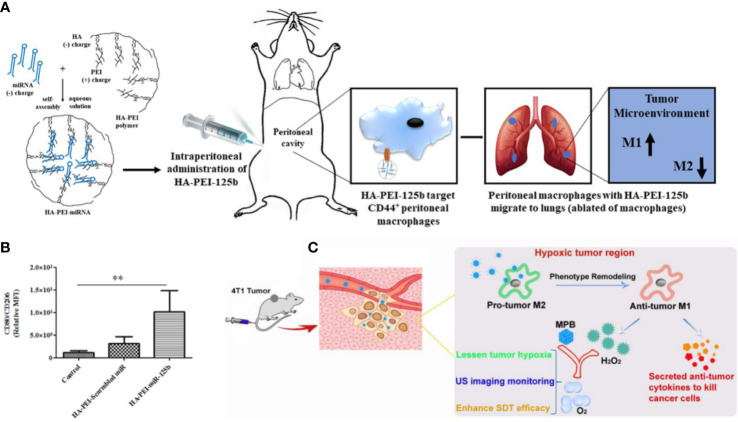
CD44 biomarker for TAM targeting and repolarization. **(A)** Assembly of HA-PEI and miRNA nanomedicine, which targets CD44^+^ TAMs to achieve a M1-phenotype repolarization. **(B)** Immuno-histochemistry of the lung reveals the proportion of CD80^+^ TAMs in TME. **(C)** LWM-HA fragments modified MBP nanomedicine effectively repolarize TAMs from M2- to M1-phenotype and produce oxygen to relieve tumor hypoxia. **(A, B)** were reproduced from ref (46); **(C)** was reproduced from ref (47).

Since CD44 receptor has been attracted as a surface marker for M2-TAM, HA-modification enabled nanomedicines to specifically recognize M2-TAM and achieve precise drug delivery. For instance, Parayath et al. constructed a poly ethylenimine (PEI)-based nanoparticle with HA decorated on the outer layer to target TAMs *via* HA-CD44 interaction (**Figure 2A**) (46). In this study, a reprogramming micro-RNA miR-125 which served as a pathologically relevant signal for initiating the phagocytotic and inflammatory function of TAMs while inhibiting the function of NF-*κ*B, was loaded into the HA-modified nanoparticles to promote M1-TAMs repolarization. The *in vivo* study showed that such nanomedicine successfully increased the level of CD80^+^ TAMs that exhibited DC-like properties by 5 folds in KRAS/p53 genetically engineered non-small cell lung tumor model (**Figure 2B**). Similarly, Zhang et al. decorated LMW-HA fragments on the surface of mesoporous Prussian blue (MPB) nanoparticles (**Figure 2C**) (47). After systematic administration, the LWM-HA-modified nanoparticles efficiently enriched into tumor site and were selectively taken up by M2-like TAMs to achieve a M2-M1 repolarization, and also served as an oxygen-generator to normalize hypoxia within the TME. The *in vivo* anti-tumor evaluation showed that LMW-HA modified MPB effectively inhibit the proliferation and metastasis of 4T1 tumors by improving TME. In addition, Kim et al. validate the TAM content by ^64^Cu-Labeled Macrin. To be specific, Marcrin was made of poly-glucose and was cross-linked by L-lysine, which was biodegradable and could target TAM by interacting with the CD44 receptors. In this study, they verified the correlations between TAM concentration with the nanomedicine taken-up capability. The results showed that PLGA-PEG nanoparticles accumulated in the TAM-sufficient tumor by 7-fold higher than that in TAM-deficient tumor, indicating that CD44 may be an ideal target for imaging-guided TAM-oriented cancer immunotherapy (48).

### CD206 inhibition

CD206 is a membrane-bound protein consisting of distinct extracellular structural domains, a transmembrane fragment and a cytoplasmic tail, and is normally expressed on macrophages and dendritic cells (49). CD206 can recognize and bind to a variety of ligands such as peptide hormones, lysosomal hydrolases and mannose and their derivatives, and is regulated by these ligands to initiate its clearance process (50). Although CD206 is involved in endogenous molecular clearance, antigen presentation, and regulation of cellular activity, but CD206 alone cannot induce phagocytosis which requires a synergy with other receptor (*e.g.*, TLR)-induced signal (51).

The conformational changes of CD206 that occur upon ligand binding have recently been exploited as a new target for precise M2-TAMs drug delivery and reprogram of TAMs from M2 to M1-phenotype. For example, precise targeting with short peptides has exhibited certain potential for intracellular delivery of therapeutically relevant molecules (53). Jaynes et al. investigated the direct binding of CD206 to a synthetic peptide (RP-182), an analog of a naturally occurring host defense peptide. They discovered that RP-182 could activate phagocytosis and autophagy in M2-TAMs, converting these cells into antitumor M1- phenotype (52). Such therapeutic efficacy is dependent on CD206. To be specific, RP-182 initiates RAC1/CDC42 activation and IQGAP1 recruitment, promotes phagocytosis and autophagy, and co-stimulates NF-*κ*B transduction which induces macrophage repolarization by secreting TNF-*α* and IL-12. Interestingly, the reeducation of TAMs also affects the expression of immune checkpoint molecules, as PD-L1 and SIRP*α* were also observed to be decreased during this process. Niu et al. developed doxorubicin-loaded PLGA nanoparticles with surface-modification of acid-sensitive moieties or mannose. Benefited from the inherent self-fluorescent capability of doxorubicin, the interaction of the nanoparticles with TAM could be further visualized using *In Vivo* Imaging Techniques. In this study, they found that nanoparticles simultaneously surface-modified with acid-sensitive moieties and mannose more efficiently accumulate in tumor than that modified with only one ligand, expanding the application scope of CD206-targeted nanomedicine (54).

### TLR receptor

TLRs are a class of pattern recognition receptors that can detect the substances derived from invading pathogens, which play essential roles in innate immunity and inflammatory responses (55). The TLR signaling cascade can regulates the expression of pro-inflammatory genes by activating STAT1 and NF-*κ*B (56). STAT1 has been demonstrated to be a key regulator of various TLR signaling pathways, while NF-*κ*B axis is an important signaling pathway that regulates macrophage polarization. It has been shown that activation of TLR4-mediated NF-*κ*B signaling pathway in TAMs is associated with tumor growth inhibition in melanoma models (57). In fact, TLR4 is expressed in a variety of cells including monocytes and macrophages. During macrophage activation triggered by bacterial LPS and lipophosphatidic acid (LTA), the upstream TLR4 triggers intracellular signaling and then activates NF-*κ*B by stimulating MY-D88, ultimately releasing cellular inflammatory factors such as TNF-*α*, IL-1*β*, IL-6, and IL-10 (58). In general, TLR/NF-*κ*B activation promotes the M1-like polarization, but such biological process highly depends on the composition of NF-*κ*B subunits. For example, NF-*κ*B p65/p50 heterodimers can enhance pro-inflammatory cytokines production to polarize TAMs into M1-phenotype, while p50/p50 homodimers result in the M2-phenotype TAMs (59, 60).

Notably, TLR4/5/7/8 and 9 are specifically overexpressed in lung cancer tissues (61). TLR4 agonists such as bacterial LPS can effectively trigger the activation of M1-phenotype TAMs-related genes by activating activator protein-1 (AP-1) and interferon regulatory factor-3 (IRF-3) cascades. It has been discovered that TLR4 signaling *via* NF-*κ*B in TAM is also associated with Tumor metastasis suppression in mouse lung cancer models (62). Similarly, other TLR agonists such as lysophosphatidic acid (LTA), R848, and CpG also display the potency to induce anti-tumor M1 macrophages (63). TLR-7 expressed in plasmacytoid dendritic cells, B cells and eosinophils and TLR-8 predominantly expressed in monocytes and macrophages have been engaged as important targets for activating innate immune responses at tumor site (64). Upon stimulation by the corresponding agonists, the myeloid differentiation factor 88(MyD88) dependent pathway is activated. The death domain of MyD88 recruits and activates the IL-1 receptor-associated kinases including IRAK4 and IRAK1, which form the protein complex to activate the I*κ*B kinase complex. Subsequently, the activated I*κ*B kinase complexes phosphorylate I*κ*B, which further induces the release of NF-*κ*B and its translocation to the nucleus, leading to the increased release of pro-inflammatory cytokines and transcription of M1-TAMs related genes.

Recently, nanomedicines carried with TLR agonists have been demonstrated to efficiently promote M1-polarization of TAMs. As shown in Rodell et al. incorporated TLR7/8 agonist R848 into a cell-permeable lysine embedded *β*-Cyclodextrin nanoparticle (CDNP) (65). The delivery of R848 into TAMs by CDNP significantly elevated the level of IL-12, then successfully inhibited tumor growth and prevented tumor recurrence. Not only that, the combination of R848 and PD-L1 antibodies further eradicated xenograft tumors in mice. In addition, TLR4 agonist also reached a similar effect on TAM reeducation after being intracellularly delivered by nanomedicines. Inspired by plant-derived extracellular vesicles, Cao et al. manufactured ginseng-derived nanoparticles (GDNPs) to achieve TAM-specific delivery and TLR4 activation (66). With its exogenous antigen-like properties, GDNPs could be naturally phagocytosed by macrophages, triggering the activation of TLR4 pathway. The *in vivo* data showed that GDNPs significantly increased the proportion of CD86^+^ TAMs, T cells and NK cells while reducing CD206^+^ TAMs, successfully delaying the growth of melanoma. Besides, TLR activation also endows TAMs with tumoricidal capacities like increased phagocytosis and antigen-presentation, which renders it an ideal co-therapy with other means of cancer therapies. Nowadays, TLR agonists are usually used as vaccine adjuvants for TAMs-targeting immunotherapy. For instance, Muraoka et al. used nano-sized hydrogel to achieve the co-delivery of long antigen peptide and TLR agonists (67). They found that upon systematic administration, the CD4/8^+^ T cell population in CT26, CMS7, and CMS5a/NY tumors was significantly elevated, and the tumor was converted to immune-sensitive status.

### STAT inhibition

STATs are a group of transcription factors that can bind to the promoters of target genes and are widely found in mammalian cells (68). Under the activation of JAK, STAT is phosphorylated and dimerized, and the dimerized STAT displays reduced affinity to its receptor and can translocate to the nucleus, where it acts as a transcription factor for regulatory gene expression (69). The JAK-STAT pathway is an inflammatory signaling pathway which can respond to both internal and external pathogens. JAK and STAT also participate into many signaling pathways that regulate cell growth, differentiation, survival, and pathogen resistance, and are associated with various cytokines release (70).

STAT signaling is important for macrophage polarization, while IFN-*γ* signaling inducing phosphorylation of downstream STAT1, which in turn activates STAT1. The deacetylation and subsequent phosphorylation of STAT1 allow it to relocate to the nucleus and further result in M1 phenotype repolarization. It is demonstrated that regulation of STAT1-mediated glycolysis could promote M1 macrophage polarization (71). Besides, STAT1 can bind to another important pro-inflammatory transcription factor, IRF5, which also effectively converts TAM to M1 type (72, 73). Therefore, STAT1 may be a potential target for reeducating TAM to M1-phenotype. In contrast, IL-4 and IL-13 induce the polarization of macrophages to M2 phenotype through the JAK-STAT pathway. These two cytokines induce the activation of STAT6, which is necessary for the polarization of M2-like TAM (73, 74). IL-4 activates JAK by binding to IL-4R*α* on the membrane leading to receptor dimerization, which causes JAK kinases coupled to the receptor to aggregate with each other and phosphorylate JAK (74). Subsequently, JAK activates growth factor receptor bound-2 and PI3K, which recruit STAT6 and phosphorylate it into the nucleus. The phosphorylated STAT6 can bind directly to KLF-4 and PPAR-*γ*, promoting M2 polarization of macrophages by initiating gene transcription or acting on transcription factors interferon regulatory factor 4 (75).

**Figure 3:**
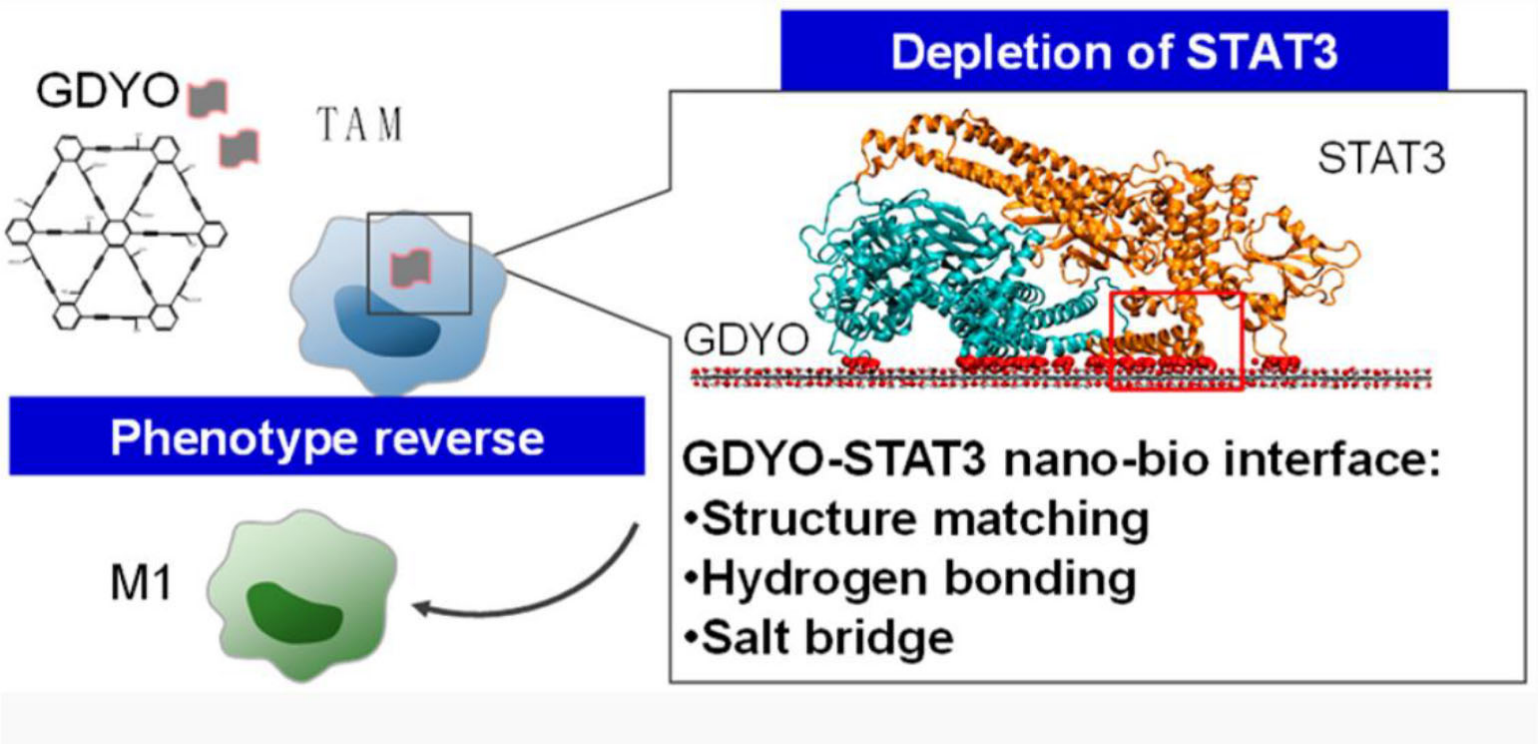
The GYDO-nanosheet in TAMs binds the surface of STAT3 to hinder its function, thereby reeducating TAMs towards M1-like pro-inflammatory phenotype. Reproduced from ref (76).

To achieve TAM-targeting regulation on STAT pathway without inducing systematic toxicity, a two-dimensional carbon-based nanomaterials-graphdiyne oxide (GDYO) nanosheets was designed to realize precise macrophage repolarization regulation (**Figure 3**) (76). With the help of structure matching and molecule dynamics, the prepared nanoscale sheet well bound to STAT3 and prevented its entry into the nucleus. The STAT3-DNA interaction is hindered, which then effectively reversed the immunosuppressive properties of TAMs. The *in vivo* data showed that the elevation of M1-TAMs level further increased the CD8^+^ T cell to regulatory T cell ratio by five-fold after *i.p.* administration, thereby dramatically inhibiting the tumor growth.

### HDAC inhibition

Histone deacetylases (HDAC) are a group of functional enzymes that exhibit the capability of removing acetyl groups from lysine residues, leading to alterations in DNA transcription through transcription factors (77, 78). HDAC is important for both the immune pro-inflammatory response and the maintenance of body homeostasis. HDACs are divided into four classes, *i.e.*, class I HDAC (HDAC 1, 2, 3 and 8), class II HDAC (HDAC 4, 5, 6, 7, 9 and 10), class IV (HDAC 11) and class III (sirtuin family: sirt1-sirt7). Class II HDACs are divided into subclasses IIa (HDACs 4, 5, 7 and 9) and IIb (HDACs 6 and 10). Among them, HDAC3 is an important pro-inflammatory effector that plays a role in various inflammatory responses by blocking the NF-*κ*B signaling pathway, and HDAC5 is associated with the activation of NF-*κ*B signaling pathway (79). HDAC7 and HDAC9 can move between the nucleus and cytoplasm to regulate signaling and gene expression, which allows them to interact with other signaling pathway proteins and thereby regulate the polarization of macrophages (80). HDAC7-u, a specific homotype of HDAC7, acting as a positive regulator of the TLR response in macrophages, promoted the production of IL-6, IL-12, p40 and TNF-α to a lesser extent (81). HDAC9 is highly expressed during macrophage differentiation. Deletion of HDAC9 resulted in increased transcription of ABCA1, ABCG1, and PPAR-*γ* in macrophages, which further resulted in a shift of macrophages from proinflammatory M1 to anti-inflammatory M2 phenotype through the action of PPARγ (77). These results broadly suggest that HDAC9 plays an important role in the regulation of macrophage function. In addition, explicit inhibition of monocyte responses to M-CSF and GM-CSF by class IIa HDAC inhibitor may also illustrate the promotional effect of HDAC on CSF1-CSF1R pathway, which provides an alternative way to regulate signaling pathways for macrophage polarization (81, 82).

**Figure 4:**
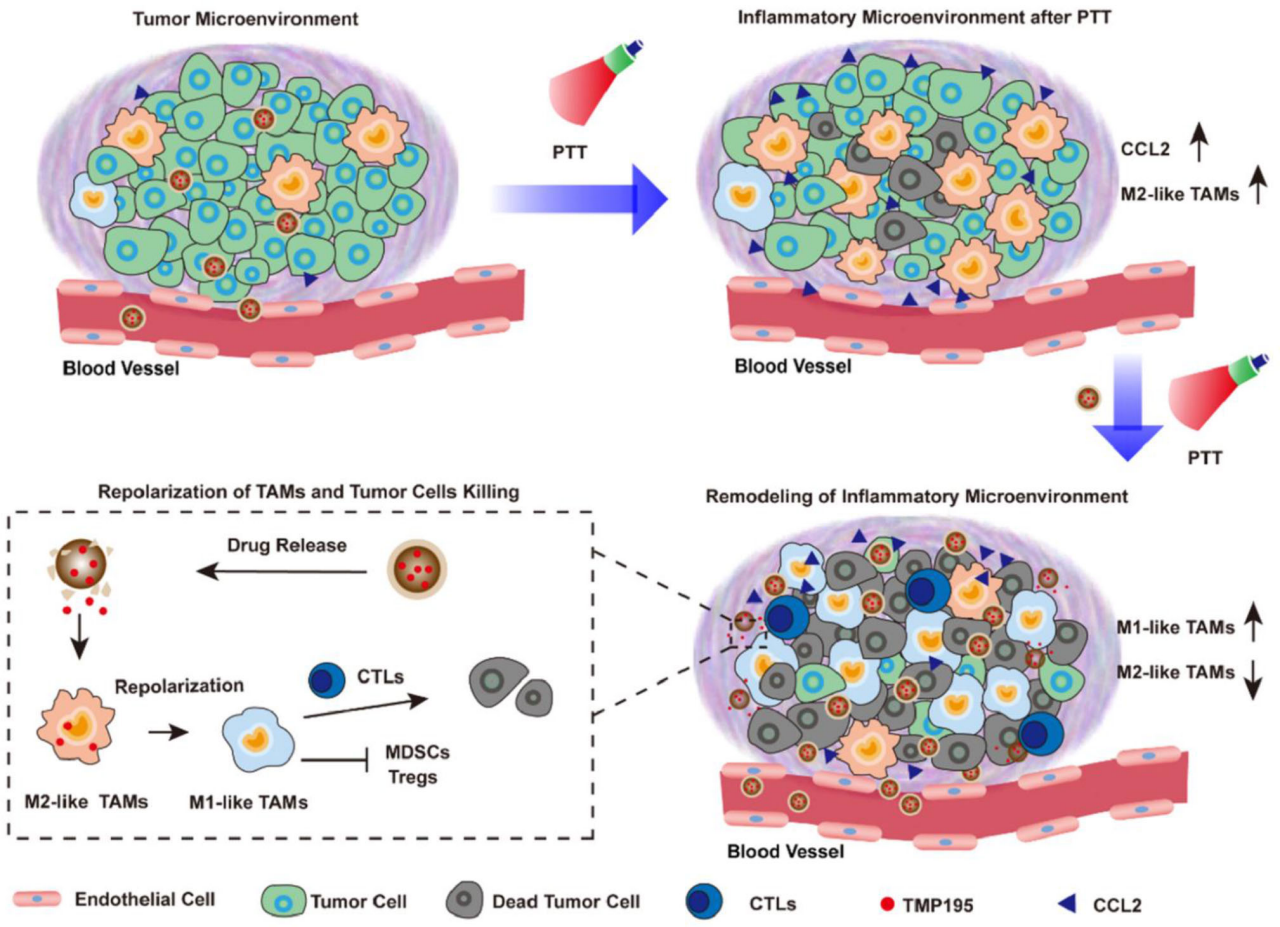
Nanoparticles incorporating HDAC inhibitor TMP195 was used for post-photothermal therapy to reeducate M2-like TAMs in the TME for better prognostics, reproduced from ref (83).

With these regards, Yue et al. designed a biomimetic nanoparticle that incorporated HDAC inhibitor TMP-195. (**Figure 4**) (83) TMP195 could compete for binding to their acetyl-lysine binding sites, resulting in the transcriptional inhibition of IIa HDACs. And TMP195 treatment significantly reduced the proportion of tumor-promoting TAM (marked by CD206). In addition, they found that most TAMs after photothermal therapy exhibited M2-like properties, which may contribute to cancer recurrences. As a result, 60% of mice in the nanoparticle plus PTT group achieved tumor eradication, while none of the other groups could completely inhibit tumor progression. Vorinostat, the first FDA-approved HDAC inhibitor, has been shown to inhibit intra-tumour TAM infiltration. Peng et al. demonstrated that vorinostat could repolarize M2 macrophages into M1 and overcome gefitinib tumor resistance. They co-delivered vorinostat and gefitinib to CD206-positive M2 TAM in the liposome-based formulation. After the treatment, the mRNA expression levels of M2 macrophage-associated markers such as Il-10 and CD206 decreased, while the expression of M1 markers including CD86 and TNF-α increased. As a result, such therapeutic combination achieved 70% tumor inhibition (84).

### cGAS-STING activation

The cGAS-STING signaling pathway plays an essential role in the innate immune system, whose function is to detect the presence of cytoplasmic DNA and initiate the expression of inflammatory genes in response. GMP-AMP Synthase (cGAS) is a pathogen recognition receptor that has received attention for a long time. During pathogen entry and proliferation within the host cell, pathogen DNA is released into the cytoplasm of the host cell and accumulates therein. At the same time, the invading pathogen also damages the host cell, resulting in the release of its nuclear and mitochondrial DNA into the cytoplasm. All the free cytoplasmic DNA derived from both self and exogenous sources will then be efficiently recognized by the cGAS and leads to a conformational change in the active site of cGAS, resulting in cGAS activation. cGAS then catalyzes the chemical reaction between intracytoplasmic GTP and ATP to form Cyclic guanosine-phosphate adenosine (cGAMP) molecules. As a messenger of the innate immune system, cGAMP binds to STING (stimulated interferon gene), a key component of the cellular immune response to abnormal cytosolic DNA, resulting in a conformational change and activation of STING (85). STNG can further recruit and activate TBK1 kinase and IKK kinase, which in turn activate the downstream IRF3 and NF-*κ*B, thereby strongly upregulate the expression of type I interferon and a series of inflammatory factors and potentiating the immune response (86).

In TME, TAMs are the major cells in which the cGAS-STING signaling pathway is activated. An increasing number of studies have found that activation of the cGAS-STING signaling pathway in TAMs is associated with TAMs polarization (87). For example, a lack of LC3-associated phagocytosis activates the STING signaling pathway through the production of self-antigens (dsDNA), which induces pro-inflammatory gene expression (IL-6, TNF-*α*) to polarizes TAMs into antitumoral M1-TAMs and inhibits tumor growth (88).

**Figure 5:**
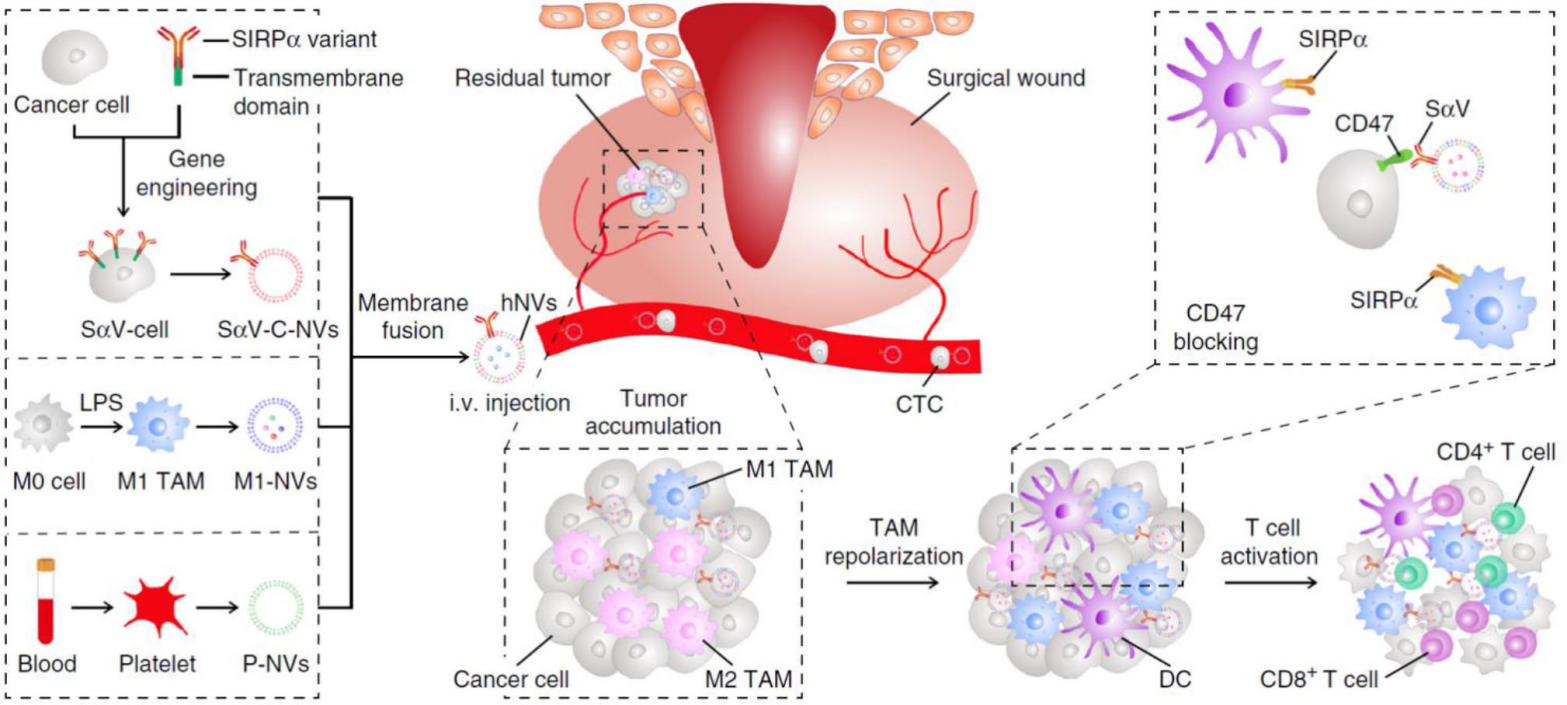
The hNVs consist of S*α*V-C-NVs, M1-NVs, and P-NVs, they could efficiently interact with CTCs in the blood, accumulate in the post-surgical site, repolarize TAMs towards M1 phenotype, and block the CD47-SIRP*α* ‘don’t eat me’ pathway to promote phagocytosis, eventually boosted antitumor T cell immunity, reproduced from ref (90).

STING agonists has been proved to be effective for TAM repolarization. Cheng et al. incorporated classic STING pathway activator cGAMP to reeducate TAMs and remodel the TME in triple-negative breast cancer (TNBC) model (89). The GAMP containing nanoparticles (GAMP-NP) improved the solubility and bioavailability of cGAMPs. The *in vitro* experiments showed that GAMP-NPs were capable of repolarizing mouse and human macrophages into M1-like phenotype and potentiate their DC-like characteristics. In combination with PD-L1 monoclonal antibodies, the GAMP-NPs effectively inhibited growth of both primary and newly inoculated tumors. Moreover, Rao et al. utilized macrophage-derived hybrid cell membrane nanovesicles (hNVs) with SIRP*α* attaching to their surfaces, which could bind macrophage-specific molecule CD47 to achieve TAM-targeted delivery of cGAMPs (**Figure 5**) (90). hNVs were found to gather in tumor site upon systemic administration in both xenograft mouse models and recurrence mouse models, while their accumulation in spleens and livers were much lower than normal liposomes. Then the SIRP*α* on hNVs’ surface blocked CD47 on cancer cells to reduce their immune escape while some hNVs were internalized by TAMs and released cGAMP to reeducate TAMs. qPCR analysis showed that genes related to the activation and recruitment of T cells were upregulated, promoting circulating T cells infiltration into the solid tumors. The *in vivo* data also showed that hNVs successfully activated M1-TAMs in TME and triggered potent immune responses upon *i.v.* injection against 4T1 tumor recurrence and metastasis.

### CSF1-CSF1R pathway

Colony Stimulating Factor-1(CSF-1), also recognized as colony-stimulating factor (M-CSF), is a cytokine considered a major growth factor regulating the production, differentiation and function of TAM (91). CSF-1 is associated with tumor progression, metastasis, angiogenesis, and treatment resistance. CSF-1 engagement with its receptor CSF-1R promotes human monocyte differentiation into macrophages, resulting in increased level of TAMs in TME to promote tumor invasion, metastasis, and angiogenesis (92). It was shown that increased expression of CSF-1 was one of the characteristics of increased tumor aggressiveness and was associated with poor prognosis of cancer patients (93). CSF1 is closely associated with the polarization of the M1- to M2-phenotype. Interaction of CSF1 with CSF-1R triggers the autophosphorylation of multiple intracellular tyrosine residues, which leads to the initiation of a series of phosphotyrosine-based signaling cascades and the upregulation of PLC-*γ*2, STAT3, and Erk1/2 and resulting in the subsequent nuclear localization of the transcription factor Sp1 promotes the differentiation of macrophages towards M2-phenotype (94).

Considering its biological functions, blocking CSFR/CSF axis has been an alternative strategy to reduce TAM infiltration. Kulkarni et al. explored the antitumor therapeutic efficacy though inhibiting CSF-1R (95). Dual-blockade of CD47/SIRPα and MCSF/CSF-1R axis was achieved by supramolecule was assembled from clinically available small molecule AK750 to interfere with CSF-1R signaling pathway and displayed SIRP*α* antibodies to target TAMs. After treating with the supramolecule, elevated CD86 expression was observed in macrophages, confirming the repolarization of TAMs from M2 into M1-phenotype. In B16/F10 melanoma and 4T1 breast cancer models, the supramolecule also exhibited potent antitumor and anti-metastatic efficacy. Similarly, Ramesh et al. designed a PC/DSPE-PEG-based nanoparticle loaded with CSF1R and MAPK inhibitors (96). When the nanoparticles were internalized by M2-like TAMs, CSF1R and its downstream protein ERK were significantly downregulated, suggesting robust inhibition of the CSF1/CSF1R pathway. Consequently, TAMs were reeducated toward M1-like TAMs and the nanoparticle showed excellent tumor suppressing activity in 4T1 tumor mouse model. Apart from CSF1R inhibition, CSF1 depletion also demonstrated strong potency when applied with photodynamic therapy. Chen et al. designed photosensitizer-conjugated TAM membrane-derived nanoparticles (NPR@TAMM) to achieve the depletion of CSF1 (97). Since the membrane of TAMs are abundant with CSF1R, the nanoparticle could consume CSF1 in the TME without causing immunosuppressive events. At the same time, photodynamic therapy also created an inflammatory environment in the TME and induced immunogenic cell death. Together with the TAM-reeducating nanoparticles, M1-like TAMs attracted antigen-presenting cells and tumor-specific effector T cells, resulted in robust antitumor responses.

## Conclusion and perspectives

TAMs are one of the most important immune cells within the TME, which is the most abundant tumor infiltrating cell in immune-deserted and immune-excluded tumor phenotypes and carrying out important anti-tumor functions in inflamed tumors. The double sword mechanisms of TAMs are gradually understood with the help of advancing basic biology on macrophages. Nowadays, it is inappropriate to divide TAMs in a binary M1-M2 categories. Under different stimuli, the characteristics and behaviors of TAMs are versatile and complicated. In this context, we believe it is necessary to concentrate our focus on the exact targets and the signaling pathway responsible for the TAM behavior regulation. The pro-tumorigenic and anti-tumor properties of different TAM phenotypes stems from its highly plasticity. Reeducating TAMs toward a tumoricidal phenotype rather than simply deplete TAMs could be a more effective strategies for turning foes to friends when fighting with cancer. The phagocytosis and antigen-presenting potential of TAMs can also assist priming tumor-specific T cells and eliciting robust anti-tumor immune responses when under proper environmental education, including abovementioned TLR activation, STING activation, CD44 blockade, CD206 blockade, STAT inhibition, HDAC inhibition, and CSFR blockade. Among them, CD44, CD206, and CSFR are TAM surface molecules that can be utilized as targets to achieve TAM-specific delivery and their activation or inhibition alone can influence the behavior of TAMs. Their ligands are ascertained, and the developments of artificial ligands with superior properties are under active research. For these ligands, they can be easily attached to the surface of the intended nanomedicine or can be released extracellularly for following interaction with the corresponding receptors. However, in most preclinical studies, inhibition of CD44 or CD206 alone is not enough to attain the required intensity for TAMs reeducation. And so currently they are regarded as targeting units for nano-based strategies until more effective ligands are developed. On the other hand, CSF depletion or CSFR inhibition alone could already realize significant reeducation of TAMs. Various strategies and co-therapies have been designed accordingly. As for TLR, STING, STAT, and HDAC, they are classic intracellular pathways for cancer therapies, and various agonists and inhibitors have been developed in the past decades. Since these pathways are common in human cells, corresponding strategies for these pathways usually focus on the TAM-specific delivery to minimize their off-target side effect and augmenting the efficacy of TAM reeducation. To fully realize the therapeutic potential, their roles as combinations with other cancer therapies are appreciated for fully disclosure.

Years of work have been dedicated to studying the role and function of TAMs, as well as exploring TAMs-associated anti-tumor strategies. Various forms of TAM-targeted delivery platforms exhibited their unique advantages and disadvantages. To attain the precise modulation of TAMs, ideal therapeutic strategies should be capable of 1) realizing specific delivery towards TAMs, 2) achieving high efficiency for drug internalization by TAMs and 3) achieving sustained drug release and continuous normalization of TME (98). Therefore, nanomedicines have showed great potential because of their flexibilities of design. Targeting ligands such as HA, mannose can be attached to the surface of nanomedicines for targeting CD44 and CD206 molecules on TAMs. By utilizing the phagocytotic ability of TAMs, nanoparticles can be rapidly internalized and release their payloads within the cells. Their payloads, being protected by the nanomaterials, is secure from biodegradation. As a result, a wide variety of therapeutic agents can be employed in order to achieve different modulatory outcomes, such as small molecules, antibodies, nucleic acids, and even immunomodulatory metal ions (99). Moreover, nanomedicines can synergize with other approaches such as phototherapies, sonodynamic therapies, and radiotherapies to broaden their applicability in both preclinical research and clinical trials. Besides TAM modulation, the design of TAM-targeted nanomedicine and identification of TAM biomarkers also inspired the design of TAM imaging strategies, which can provide the information for tumor diagnosis, defining the status of TME and TAM recruitment, and evaluating the efficacy of treatment (48, 54, 100). Specific design of nanomedicine can even achieve diagnosis and therapeutic functions simultaneously. Through revealing the relationship between the TAM content and the eventual therapeutic outcome, we can gain a deeper understanding of the biological roles of TAM in tumor progression and the effectiveness of the targeting modulation therapeutic strategies (101). The development of TAMs-directed imaging technology further facilitate the research on TAM-reeducation and optimize the existing treatment modalities.

With the in-depth understanding of TAM repolarization mechanism and its phagocytic function, TAM-based nanomedicines have been promising tools for efficient cancer immunotherapy. However, there are some issues still need to be addressed. For instance, as the boundary between M1 TAMs and M2 TAMs is indistinct, the markers indicating immunosuppressive TAMs need to be explored for targeted delivery. The effects of regulation on different signaling pathways of TAMs is still not thoroughly clear, which requires the future efforts to focus on understanding the behavior of TAMs under different environmental regulation. Besides, the complicated components of current nanomedicines greatly hinder their clinical or industrial application, thus the preparation of nanomedicines that are simpler and capable of large-scale production is crucial for the development of TAM-targeted drug formulations in clinical practice. In addition to traditional nano-delivery systems, some emerging delivery strategies like bacteria-based platforms and cell-based platforms are still seldom applied for TAM modulation. These platforms with distinct characteristics might interact with TAMs in brand new mechanisms that might result in better immunotherapeutic efficacy (102, 103). We anticipate the cutting-edge nanomedicines-based TAM-targeted regulation strategies hold great potential for future cancer immunotherapy.

## Author contributions

XYS, CJZ, and LXZ conceived the topic of the Review. XYS, SCZ, YDY, TH, ZX, JZ, CJZ, LHZ, and LXZ, contributed to discussions of the content. XYS, SCZ, and CJZ co-wrote the manuscript. XYS, CJZ, JZ, LHZ, and LXZ corrected and reviewed the article before submission.

## Funding

We acknowledge the financial support of the National Natural Science Foundation of China (32101123), Zhejiang Provincial Natural Science Foundation of China (LTY21H160001), Natural Science Foundation of Ningbo (Grant 20221JCGY010704 and Grant 2021J320), and the authors of the primary studies.

## Conflict of interest

The authors declare that the research was conducted in the absence of any commercial or financial relationships that could be construed as a potential conflict of interest.

## Publisher’s note

All claims expressed in this article are solely those of the authors and do not necessarily represent those of their affiliated organizations, or those of the publisher, the editors and the reviewers. Any product that may be evaluated in this article, or claim that may be made by its manufacturer, is not guaranteed or endorsed by the publisher.
